# Health Status Recognition of Yellow-Feathered Broilers in Floor-Rearing Environments via Whole-Body and Comb Feature Fusion

**DOI:** 10.3390/ani16162559

**Published:** 2026-08-17

**Authors:** Jialei Xue, Tian Hua, Hongliang Guan, Hao Bai, Guobin Chang, Bin Li

**Affiliations:** 1 College of Information and Artificial Intelligence, Yangzhou University, Yangzhou 225009, China; dx120230100@stu.yzu.edu.cn (J.X.); msghl@outlook.com (H.G.); 2 College of Animal Science and Technology, Yangzhou University, Yangzhou 225009, China; huatian0202@163.com (T.H.); gbchang1975@yzu.edu.cn (G.C.); 3 Joint International Research Laboratory of Agriculture and Agri-Product Safety, The Ministry of Education of China, Institutes of Agricultural Science and Technology Development, Yangzhou University, Yangzhou 225009, China; bhowen1027@yzu.edu.cn

**Keywords:** yellow-feathered broiler, broiler health monitoring, floor-rearing system, computer vision, deep learning, transformer, feature fusion

## Abstract

In commercial floor-rearing environments for yellow-feathered broilers, visual screening of broilers with observable health-related abnormalities is challenged by low illumination, individual occlusion, and the limited use of local comb information. This study proposes a two-stage global–local framework for yellow-feathered broilers. Images were collected from one commercial floor-rearing farm; after screening, 1463 images were used for key-region localization, and 2031 samples were constructed for health-status recognition. Samples were labeled as Healthy or Unhealthy by expert consensus according to observable phenotypic characteristics, rather than veterinary-confirmed disease diagnoses. First, a Dual-Target YOLO (DT-YOLO) model localized whole-body and comb regions. Second, a Transformer network integrated features from paired or single available inputs for classification. DT-YOLO achieved a mean Average Precision (mAP) of 97.56% at the 0.5 IoU threshold. For paired whole-body–comb inputs, the proposed classification model achieved an Accuracy of 97.11%, Macro-Precision of 97.38%, Macro-Recall of 96.58%, and Macro-F1 of 96.95%. Under the mixed-input condition, the model maintained an Accuracy of 96.06% and a Macro-F1 of 96.02%. These findings indicate that combining whole-body and comb information can support visual screening of unhealthy broilers under the single-farm floor-rearing conditions represented in the current dataset. Its applicability to other farms, breeds, and management conditions requires independent external validation.

## Simple Summary

Computer vision may assist the visual screening of broilers showing observable health-related abnormalities in commercial farming environments. This study developed a two-stage deep learning framework for yellow-feathered broilers using images collected from one commercial floor-rearing farm. The method first localized the whole-body and comb regions and then integrated global and local visual information for health-status classification. The proposed framework showed improved recognition performance when whole-body and comb information were jointly considered. These findings suggest that combining the two regions may support visual screening of unhealthy broilers under the evaluated conditions. However, the labels reflected observable phenotypic abnormalities rather than veterinary diagnoses, and validation using independent data from additional farms is needed before broader use can be considered.

## 1. Introduction

According to statistics from the China Animal Agriculture Association, the annual production of broilers in China reached 16.2 million tons in 2025 [1]. Among various rearing systems, floor-rearing has been widely adopted in broiler production because it better aligns with animal welfare principles [2]. However, the continuing expansion of floor-rearing operations has made individual-level health monitoring increasingly challenging, potentially delaying the identification of unhealthy broilers [3]. In this study, the term “unhealthy broilers” refers to broilers exhibiting visually observable signs suggestive of compromised health rather than those with a veterinary-confirmed diagnosis. Such broilers may exhibit reduced activity, ruffled plumage, abnormal postures, and changes in comb appearance, including pallor, apparent shrinkage, or drooping [4]. However, these observations are nonspecific, and comb color and morphology may also vary with age, sex, genotype, environmental conditions, and stress. Previous veterinary research indicates that the comb is androgen-dependent and may function as a fitness-related trait associated with disease resistance, health, and nutritional status [5]. Therefore, when evaluated in conjunction with activity level, posture, and plumage condition, comb phenotype may provide useful, non-invasive information about the overall health status of broilers but should not be regarded as definitive diagnostic evidence of disease. If unhealthy broilers carry transmissible pathogens, infection may spread rapidly throughout the flock, thereby threatening production safety and causing considerable economic losses [6,7]. Therefore, the early identification and timely intervention of unhealthy chickens are of great importance for reducing disease transmission risks and ensuring farming profitability. At present, unhealthy chicken detection in commercial production mainly relies on manual inspection. Farmers typically make comprehensive judgments based on phenotypic characteristics [8], posture features [9], behavioral patterns [10,11], vocal features [12], and fecal characteristics [13]. However, this approach is highly subjective and prone to both false detections and missed detections. In addition, the inspection process is labor-intensive and time-consuming, while its accuracy and efficiency largely depend on the experience and professional expertise of farm personnel [14].

In recent years, with the rapid development of computer vision and deep learning technologies, intelligent vision-based methods for non-contact automatic recognition of chicken health status have attracted widespread attention [15]. Compared with traditional manual inspection, these methods enable continuous, objective, and efficient monitoring under natural chicken activity, effectively reducing false positives and missed detections caused by human subjectivity. Existing whole-body approaches can be broadly divided into two categories according to their recognition targets. The first category focuses specifically on locomotor disorders. Aydin et al. [16] identified lame broilers using movement-related variables, including walking speed, step frequency, and stride length, whereas Nasiri et al. [17] combined Convolutional Neural Network (CNN) and Long Short-Term Memory (LSTM) models to capture temporal posture and gait patterns for lameness detection. These methods primarily assess locomotor impairment and generally depend on visible movement trajectories. The second category addresses broader visual abnormalities associated with compromised health. Zhuang et al. [18] combined broiler posture features with a support vector machine for health-status classification, while Zhuang et al. [19] developed an improved feature-fusion Single Shot MultiBox Detector to extract whole-body appearance features, including feather texture and posture, for diseased-broiler recognition. Cui et al. [20] further improved YOLO-v5 to identify unhealthy broilers from whole-body phenotypic characteristics in complex caged environments. Taken together, these studies demonstrate the value of whole-body phenotypic information for automated broiler health monitoring. However, their recognition targets differ: lameness and gait studies identify specific locomotor abnormalities, whereas general health-monitoring studies identify unhealthy broilers based on nonspecific visual signs of compromised health without determining a particular disease or establishing a veterinary diagnosis.

In addition to whole-body phenotypic characteristics, local key regions can also provide important cues for assessing chicken health status [21], and existing local-region approaches can be further categorized into comb-focused and head-region-based methods. At the most localized level, Bakar et al. [22] used comb chromaticity as a standalone feature to distinguish infected from healthy chickens, demonstrating its potential for non-invasive health monitoring. At the head-region level, Ansarimovahed et al. [23] used YOLO-v8 to localize chicken heads in infrared images and combined color and texture features to detect avian influenza and Newcastle disease, achieving accuracies of 92.48% and 94.20%, respectively. Guo et al. [24] incorporated phenotypic features of the comb, eyes, and wattles into an improved YOLOv11 model to recognize four predefined poultry diseases, achieving a mean average precision of 97.2%. Bi et al. [25] combined comb texture features with geometric features of the pupil and subsequently used a support vector machine for broader diseased-versus-healthy classification. Collectively, these studies demonstrate the value of local head and comb information, although their recognition targets and feature scopes differ. Some head-region methods focus on predefined diseases, whereas others identify broader visual abnormalities using multiple head features. In contrast, comb-focused methods primarily analyze isolated color or texture information. Although these local features can reveal visually salient abnormalities, they are generally modeled without the complementary posture, plumage, and overall appearance information provided by the whole body.

Based on the studies reviewed above, the explicit joint modeling of global whole-body phenotypes and fine-grained comb characteristics appears to have received comparatively limited attention, particularly for the general visual identification of unhealthy broilers under floor-rearing conditions. Whole-body models can capture posture, plumage condition, and overall appearance but may overlook subtle abnormalities in local regions. Conversely, head- or comb-based models provide detailed local information but generally lack the broader phenotypic context required to interpret these nonspecific signs. Moreover, gait-based methods and disease-specific models address more narrowly defined recognition targets and are therefore not directly equivalent to general visual health screening. These considerations are particularly relevant in floor-rearing environments, where inter-individual occlusion can result in incomplete whole-body information, while low-light conditions can impair the extraction of color and morphological features from the relatively small comb region. Consequently, integrating complementary whole-body and comb information while accounting for occlusion and illumination variation may provide a more comprehensive basis for the general visual assessment of broiler health status.

To address these challenges, this study proposes a two-stage global–local feature-fusion framework for the visual recognition of health status in yellow-feathered broilers. In the first stage, a Dual-Target YOLO (DT-YOLO) model simultaneously localizes the whole-body and comb regions of each broiler. Based on the localization results, the second stage constructs paired whole-body and comb images and employs a Transformer-based classification network [26] to integrate their complementary representations for the classification of healthy and unhealthy broilers.

The main contributions of this study are summarized as follows:

- A two-stage health-status recognition framework is proposed for the general visual identification of healthy and unhealthy yellow-feathered broilers in practical floor-rearing environments. The framework combines simultaneous localization of the whole-body and comb regions with global–local feature fusion.
- A key-region synchronous localization method based on Dual-Target YOLO (DT-YOLO) is developed to simultaneously localize the whole-body and comb regions. The method is designed to improve region localization and feature representation under inter-individual occlusion and low-light conditions.
- A Transformer-based health-status classification method is developed to integrate global whole-body representations with fine-grained local comb features. This design enables complementary information from the two regions to contribute jointly to the classification of healthy and unhealthy broilers.
- A health-status recognition dataset of yellow-feathered broilers is constructed under practical floor-rearing conditions. Comparative experiments using whole-body features alone, comb features alone, and their fused representation are conducted to evaluate the effectiveness and robustness of the proposed framework.

Overall, this study aimed to develop and evaluate a robust global–local feature-fusion framework and to determine whether integrating whole-body and comb features could improve visual health-status recognition compared with using either feature source alone, particularly under occlusion and low-light conditions.

## 2. Materials and Methods

### 2.1. Dataset Construction

**Figure 1:**
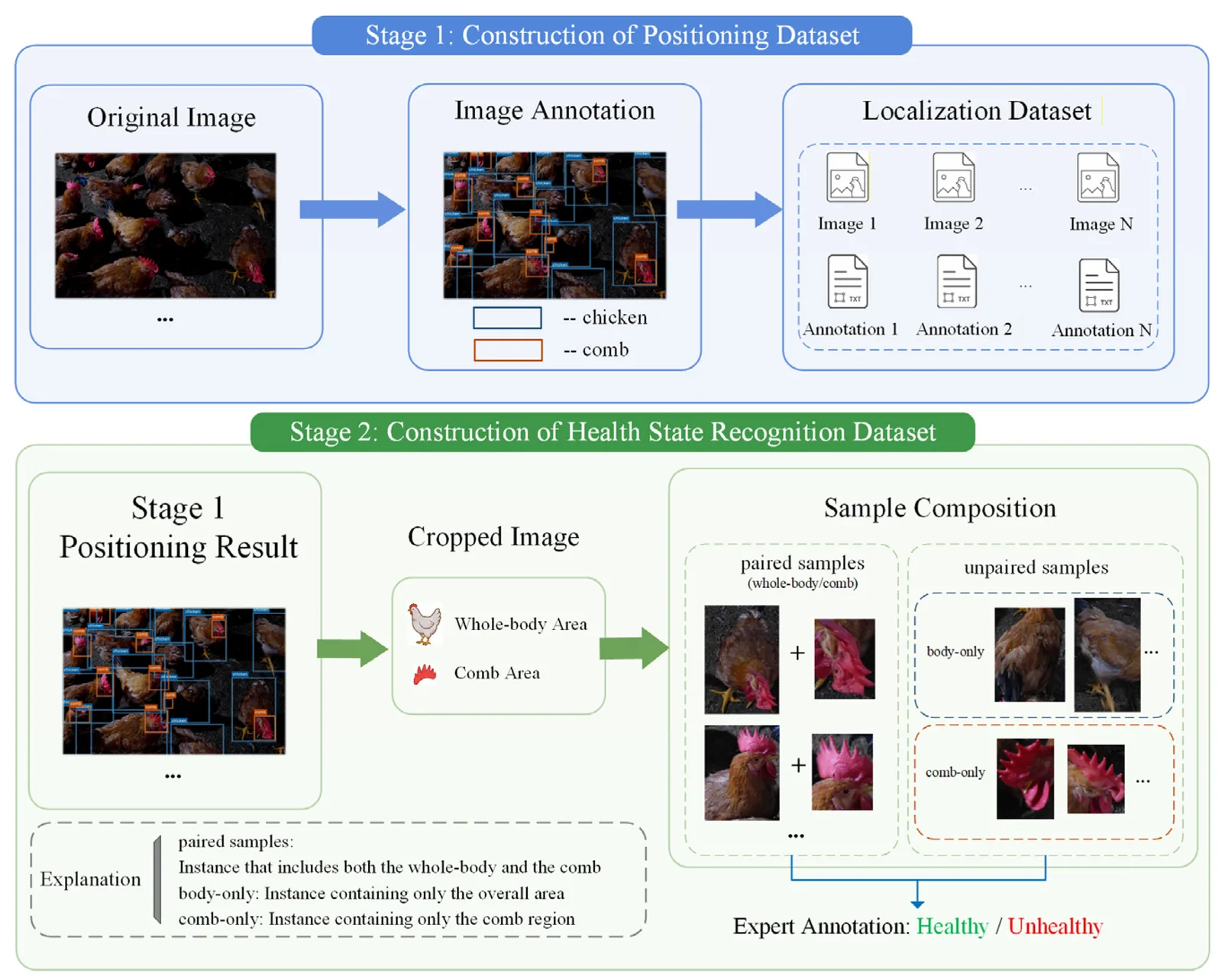
Datasets used in the two-stage method.

Two datasets were constructed for the proposed two-stage framework: a Key-Region Localization Dataset for whole-body and comb localization, and a Health-Status Recognition Dataset for instance-level healthy/unhealthy classification. The overall dataset construction workflow is illustrated in Figure 1.

#### 2.1.1. Sample Collection

Image acquisition was conducted in July 2025 at a large-scale commercial poultry farm in Gaoyou City, Jiangsu Province, China (32°50′35.9″ N, 119°40′05.0″ E). The farm used a floor-rearing system equipped with automated environmental-control facilities. The study population consisted of yellow-feathered broilers of approximately 50 days of age. Images were acquired during daytime using a Hikvision HM-TD2B28T-3/T1 industrial camera (Hikvision Digital Technology Co., Ltd., Hangzhou, Zhejiang, China) with a resolution of 1920 × 1080 pixels. The camera was mounted 1.5 m above the ground at an approximately 45° oblique downward angle. Illumination was provided primarily by natural daylight supplemented with indoor lighting. A total of 2000 raw images were collected from practical floor-rearing scenes. Images with duplicate content, severe motion blur, or unrecognizable target regions were removed during initial screening. This process yielded 1463 valid images, which retained naturally occurring challenges, including variable illumination, inter-individual occlusion, and dense flock distribution. To maintain relative flock independence during image acquisition, image collection was organized according to preassigned floor-rearing area codes. Each coded area was treated as a distinct sampling unit, and the area identifiers were retained for subsequent area-based dataset partitioning.

#### 2.1.2. Key-Region Localization Dataset

The Key-Region Localization Dataset was constructed to support the first-stage localization task. Using LabelImg software (v1.8.6), bounding boxes were manually annotated for all recognizable whole-body and visible comb regions in the 1463 valid images. Each whole-body region was assigned the class label chicken, whereas each comb region was assigned the class label comb. Regions with indistinguishable boundaries because of severe occlusion or image blur were not annotated. In total, the dataset contained 8756 chicken instances and 7468 comb instances. This dataset was used to train and evaluate the first-stage localization model for the simultaneous detection of whole-body and comb regions in floor-reared yellow-feathered broilers.

#### 2.1.3. Health-Status Recognition Dataset

The Health-Status Recognition Dataset was constructed using the output bounding boxes generated by the first-stage localization model. Whole-body and comb regions were cropped to form instance-level classification samples. To establish whole-body–comb correspondences, a spatial containment–proximity matching rule was applied. Specifically, a comb detection was considered a candidate match only when the center of its bounding box was located within a chicken bounding box. When multiple candidate chicken boxes satisfied this condition, the comb was assigned to the chicken with the smallest Euclidean distance between the centers of the two bounding boxes. The automatically generated correspondences were subsequently reviewed using an in-house semi-automatic annotation tool. Samples with severe occlusion, substantial motion blur, incomplete target regions, or uncertain whole-body–comb correspondences were excluded. To retain representative cases from practical floor-rearing environments, samples containing only a clearly visible whole-body region or only a clearly visible comb region were retained as single-part samples when the available visual information was sufficient for health-status assessment. Consequently, 2031 instance-level recognition samples were obtained, including 1630 whole-body–comb paired samples, 189 whole-body-only samples, and 212 comb-only samples.

Each sample was independently assessed by three poultry-production experts, each with more than five years of practical experience in broiler husbandry and flock health observation. The experts reviewed the original image together with all available cropped regions. The predefined visual indicators included comb pallor, apparent shrinkage, or drooping; ruffled plumage; and abnormal posture. Because these indicators are nonspecific, comb-related features were interpreted together with the available whole-body features whenever possible. Samples were labeled as Healthy or Unhealthy; the latter category denoted broilers showing visually observable signs suggestive of compromised health and did not constitute a veterinary diagnosis. A label was confirmed when at least two experts reached the same decision. Samples with insufficient visual information or unresolved uncertainty were excluded.

Approximately 100 broilers categorized as Unhealthy were repeatedly imaged, with approximately 10 images collected per bird. In contrast, healthy broilers were recorded opportunistically from unmarked, free-moving flocks; therefore, the exact number of unique healthy individuals and the precise number of repeated images per healthy broiler could not be reliably determined. Accordingly, the Health-Status Recognition Dataset was treated as an image- and instance-level dataset rather than a dataset in which each sample represented a unique individual broiler. This dataset was used to train and evaluate the second-stage health-status recognition model.

#### 2.1.4. Dataset Partitioning and Data Augmentation

Prior to data augmentation and the generation of second-stage classification samples, the 1463 valid source images were partitioned according to their preassigned floor-rearing area codes. The floor-rearing areas were treated as independent sampling units and were allocated to the training, validation, and independent test subsets at an approximate ratio of 70%, 10%, and 20%, respectively. This allocation corresponded to 1024, 146, and 293 source images, respectively. All images collected from the same floor-rearing area, together with their associated annotations and derived whole-body–comb samples, were assigned to the same subset. Therefore, repeated images of the same recorded broiler were not distributed across the training, validation, and test subsets. The Health-Status Recognition Dataset followed the same area-based partitioning principle, corresponding approximately to 1422 training samples, 203 validation samples, and 406 independent test samples. This procedure reduced the risk of information leakage caused by repeated observations of the same birds or highly similar flock backgrounds.

**Figure 2:**
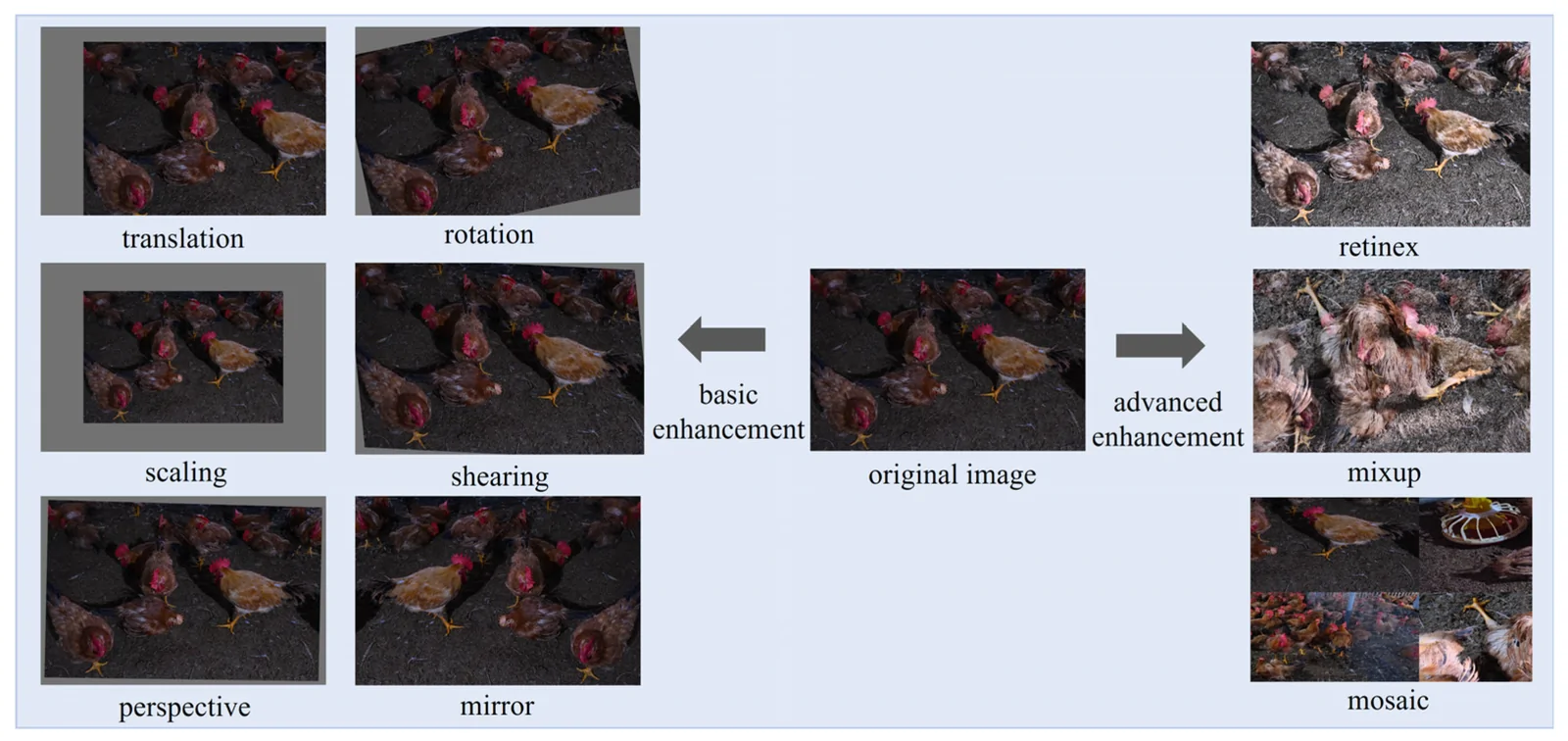
Visualization of images after basic and advanced data augmentation.

Data augmentation was performed only after dataset partitioning and was applied exclusively to the training subset of the Key-Region Localization Dataset. Neither the validation nor the independent test subset was augmented. Six basic geometric transformations, including translation, rotation, scaling, shearing, perspective transformation, and flipping, were used to simulate variations in camera viewpoint, target position, target scale, and chicken posture during image acquisition. In addition, Retinex [27], MixUp, and Mosaic were applied to improve robustness under uneven illumination, dense flock distribution, and inter-individual occlusion. Retinex was used to enhance image visibility under low-light and uneven illumination conditions. MixUp was used to linearly combine pairs of training images while retaining the corresponding annotations from both source images, thereby increasing sample diversity and reducing the risk of overfitting. Mosaic combined four training images into a single composite image, exposing the localization model to multiple targets at different scales, dense flock distributions, and simulated partial occlusion. A total of 1800 additional images were generated using the basic geometric transformations, and 900 additional images were generated using the advanced augmentation strategies. Thus, the training subset contained 3724 images after augmentation, while the validation and test subsets remained unchanged; the overall first-stage dataset contained 4163 images. To prevent augmentation-induced color bias, Retinex was applied in a class-agnostic manner only to the luminance component of images in the first-stage training set, while the chromatic components were retained. Retinex-augmented images were used only for first-stage localization, whereas the second-stage classifier was trained and evaluated using crops generated from the original, non-augmented images. Examples of the applied augmentation methods are shown in Figure 2.

### 2.2. A Health Status Recognition Framework Based on Whole-Body and Comb Feature Fusion

**Figure 3:**
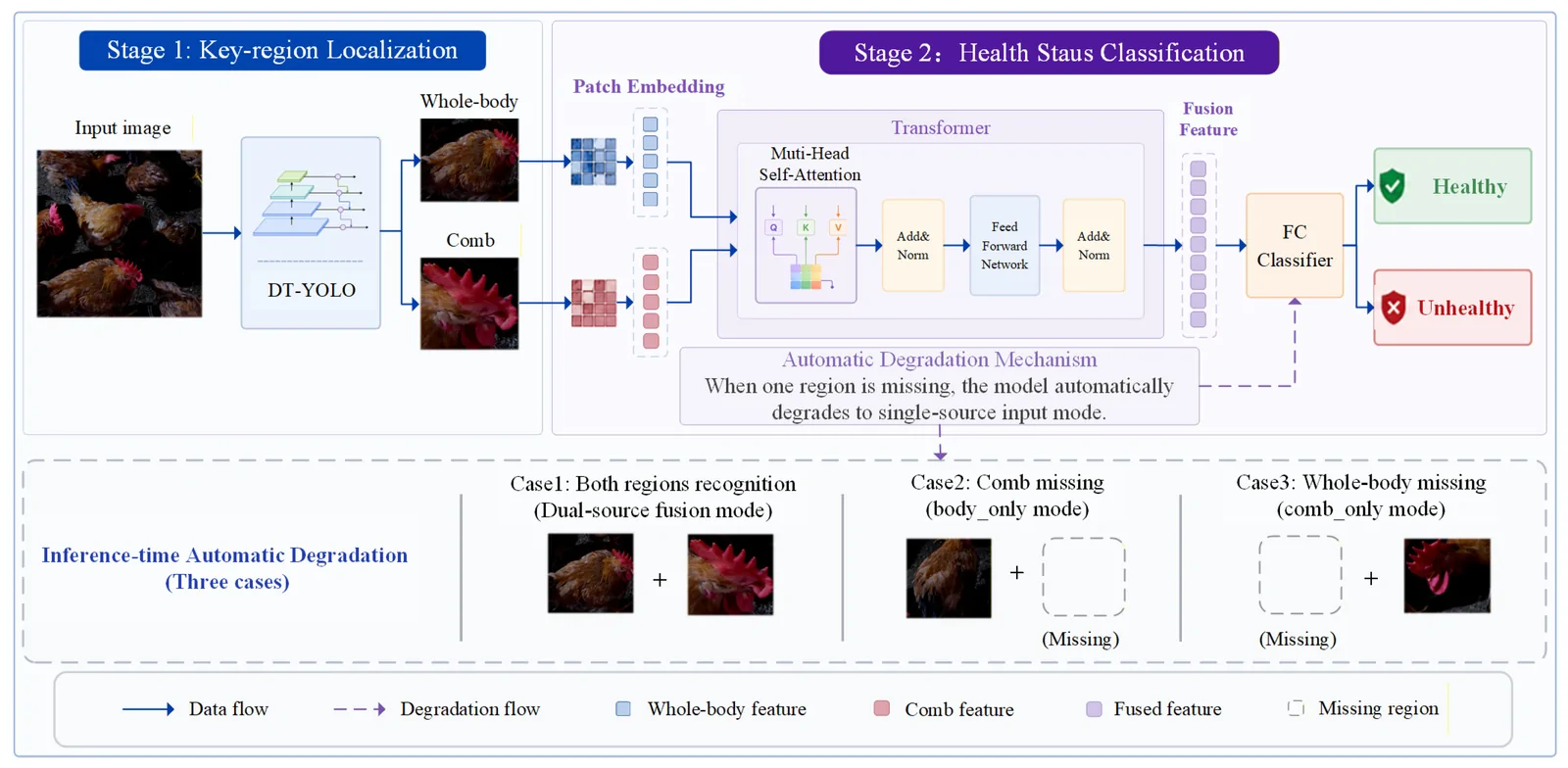
Two-stage framework for broiler health status recognition.

To address the insufficient fusion and interaction between whole-body global features and local comb features in yellow-feathered broiler health-status recognition under complex floor-rearing environments, as well as the difficulty of effectively extracting fine-grained local features caused by low illumination and individual occlusion, this study proposes a two-stage recognition framework based on adaptive fusion of whole-body and local features, as illustrated in Figure 3. From the perspective of joint global–local discrimination, the proposed framework first localizes key regions and then performs feature fusion, thereby jointly incorporating whole-body phenotypic information and local comb abnormality information into the health-status recognition process. In this way, the proposed framework improves the accuracy and robustness of chicken health-status recognition in complex floor-rearing scenarios.

The first stage is the key-region localization stage, which aims to accurately extract the whole-body and comb regions from raw flock images. Considering common challenges in real-world floor-rearing environments, such as low illumination and individual occlusion, a Dual-Target YOLO (DT-YOLO) localization model was constructed based on the YOLO-v8 detection framework. Specifically, the backbone network was reconstructed by introducing the Reversible Column (RevCol) concept [28] to enhance the integrity of whole-body feature representations under occlusion. Additionally, an InvSE module was designed and integrated into the neck network to improve the representation of fine-grained comb features under low-light conditions, thereby increasing the detection accuracy and robustness for both whole-body and comb regions.

The second stage is the health-status classification stage. Based on the detection results from the first stage, whole-body and comb image pairs are constructed and fed into a Transformer-based classification network. Using its self-attention mechanism, global whole-body features and local comb fine-grained features are interactively modeled and adaptively fused to achieve accurate chicken health-status recognition. To handle missing regions commonly encountered in real-world scenarios, an automatic degradation mechanism is introduced: when a key region is absent, the classification model automatically degrades to a single-data-source mode, extracting features and performing health-status recognition using only the available whole-body or comb region. This ensures model stability and adaptability under complex floor-rearing conditions.

#### 2.2.1. DT-YOLO Key-Region Localization Model for Broilers

**Figure 4:**
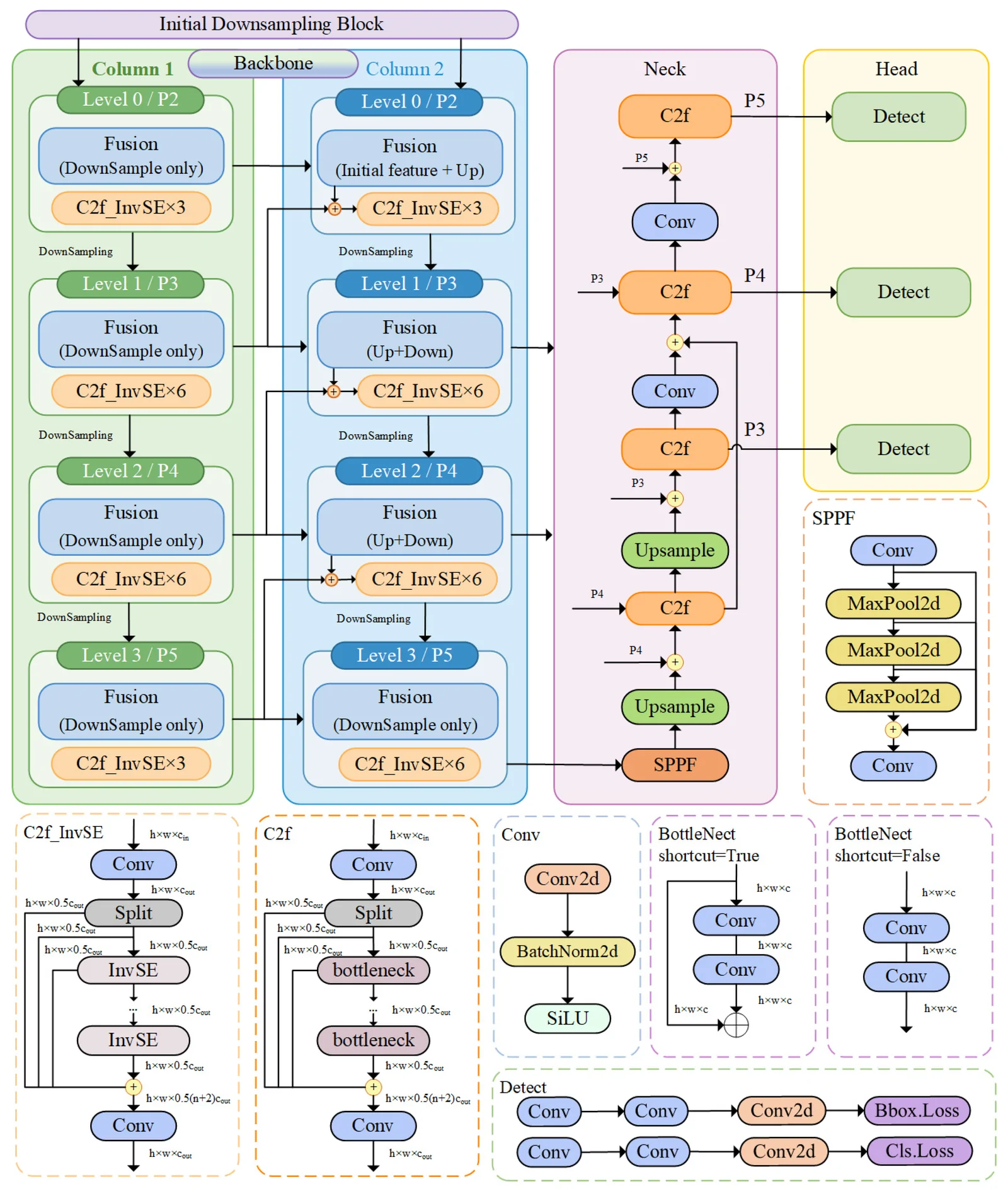
DT-YOLO network architecture.

In the first stage, the whole-body region and the comb region of the chicken are defined as two detection targets, labeled as “chicken” and “comb,” respectively. Given an input image, the key-region localization model aims to simultaneously output detection boxes for both the whole-body and comb regions, providing reliable global and local image inputs for the subsequent health-status classification stage. The overall architecture of the DT-YOLO model is shown in Figure 4. The input image first passes through the Backbone network for feature extraction, then in the Neck module, multi-scale feature fusion enhances the representation capability for targets of different scales, and finally, the detection head performs object classification and bounding-box regression, producing localization results for the two target classes: “chicken” and “comb”.

Considering that real-world floor-rearing environments commonly involve challenges such as individual occlusion and low illumination, the whole-body region of chickens often suffers from incomplete global phenotypic information due to occlusion. Meanwhile, the comb region, because of its relatively small target size and high sensitivity to low-light conditions, faces difficulties in effectively extracting fine-grained local features such as color and morphology. To address these issues, this study adopts YOLO-v8 as the baseline detection framework and further improves its Backbone network. Specifically, the RevCol reversible-column concept is introduced to reconstruct the backbone network, thereby enhancing the integrity of whole-body feature representations under occlusion conditions. In addition, the original C2f module in the Backbone is replaced with the proposed C2f_InvSE module, where InvSE stands for Inverted Residual and EfficientSE, to improve the representation capability of fine-grained comb details under low-light conditions. With these improvements, the proposed DT-YOLO model can more accurately achieve simultaneous localization of whole-body and comb regions, thereby providing reliable key-region inputs for the subsequent whole-body–comb feature fusion classification stage.

#### 2.2.2. Backbone Network Design Based on RevCol

In real-world floor-rearing environments, the whole-body region of chickens is often affected by individual occlusion and dense flocking, resulting in incomplete global phenotypic information and making accurate localization of the whole-body region more challenging. At the same time, traditional convolutional backbones have limited capability for inter-layer feature interaction during progressive downsampling, which can lead to insufficient association between local detail features under occlusion and high-level semantic features, thereby affecting the integrity of whole-body feature representation. To alleviate these issues, this study introduces a multi-column reversible structure from the Reversible Columnar Network to improve the YOLO-v8 [29] backbone, enhancing information flow and feature preservation across different hierarchical levels.

RevCol establishes cross-layer feature interaction paths across different columns and hierarchical levels, enabling shallow detail features and deep semantic features to be continuously fused during forward propagation. In this way, the integrity of whole-body feature representation under occlusion conditions can be effectively enhanced. Considering both model performance and structural complexity, RevCol was designed as a dual-column architecture in this study. On the one hand, the dual-column structure is sufficient to satisfy the requirement for cross-layer feature interaction in whole-body localization tasks under floor-rearing scenarios. On the other hand, although further increasing the number of columns may provide limited performance improvement, it could increase model complexity and computational cost.

The RevCol backbone network designed in this study is illustrated in Figure 4. Each column contains four hierarchical levels, corresponding to the P2, P3, P4, and P5 scale features in the detection framework. Each level consists of a Fusion module and several C2f_InvSE modules. Except for the first level of each column, which uses an initialization downsampling module for feature distribution, all other levels perform feature downsampling and cross-column fusion through the Fusion module. Specifically, part of the input features at the current level comes from the output of the previous level within the same column, while the other part is derived from deeper features of the previous column. This introduces richer high-level semantic information into the current level and lays the foundation for subsequent cross-column feature interaction. For the first column, since there is no auxiliary input from a preceding column, feature propagation occurs solely within the column itself, with basic feature extraction performed via progressive downsampling. The second column further integrates deep features from the first column to enhance information flow across hierarchical levels, thereby improving the integrity of whole-body feature representation under occlusion conditions.

#### 2.2.3. C2f Module Improvement Design Based on InvSE

After completing cross-layer feature fusion, each hierarchical level further performs feature extraction through the C2f_InvSE module. In the original C2f module, the Bottleneck units rely on standard convolutions for feature learning. However, the comb region typically involves small targets, which in low-light environments are prone to color distortion, weakened edges, and blurred local textures, making it difficult for the model to effectively capture fine-grained features such as comb color and morphology. To address these issues, this study proposes the InvSE (Inverted Residual and EfficientSE) module and replaces the Bottleneck units in the original C2f with it, forming the C2f_InvSE module. This design enhances the network’s ability to represent local key features of the comb region.

**Figure 5:**
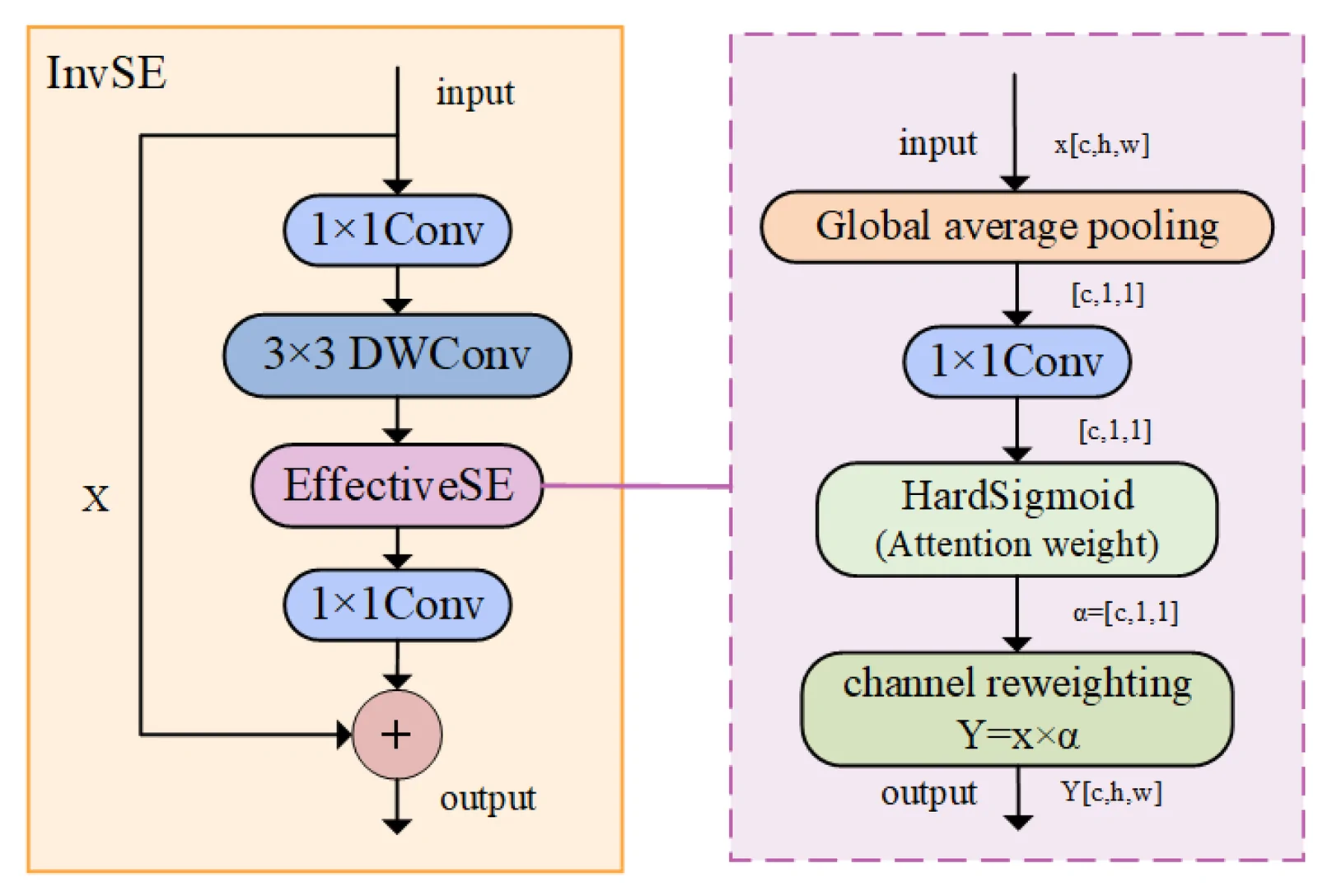
Structure of the InvSE Module.

The structure of C2f_InvSE is illustrated in Figure 5. The proposed InvSE module is designed based on the Inverted Residual architecture [30]. First, a 1 × 1 convolution is employed to expand the input channels, thereby enhancing the information representation capability in the high-dimensional feature space. Subsequently, a 3 × 3 depthwise separable convolution is adopted for spatial feature extraction, which reduces computational cost while improving the perception of fine-grained details in local comb regions. Finally, another 1 × 1 convolution is used to compress the feature maps back to the target dimension, completing the feature reconstruction process. Meanwhile, an EfficientSE attention mechanism [31] is introduced into the InvSE module for adaptive channel-wise feature recalibration. This mechanism enhances the responses of key channels associated with local comb representations, including color, morphology, and edge textures, while suppressing redundant background information caused by low-light conditions. As a result, the model’s capability for extracting fine-grained local comb features is further improved. Through these improvements, the proposed C2f_InvSE module can more effectively preserve local detail information of comb regions in complex floor-rearing environments, thereby enhancing the accuracy and robustness of key-region localization.

#### 2.2.4. Transformer-Based Broiler Health Status Recognition Model

After completing the localization of the whole-body and comb regions in the first stage, a Transformer-based health-status recognition model integrating whole-body and comb features is further constructed to identify the health status of yellow-feathered broilers. The motivation of this stage lies in the fact that, although existing methods can utilize global whole-body phenotypic features for health assessment, they still lack sufficient modeling of the relationship between local fine-grained comb features and global whole-body features, making it difficult to fully exploit their complementary advantages in health-status recognition. As an important local physiological indicator reflecting the health condition of yellow-feathered broilers, variations in comb color and morphology can provide auxiliary discriminative information for identifying unhealthy states. Based on this observation, both the whole-body region and the local comb region are jointly incorporated into the recognition model. Through joint modeling and an adaptive feature fusion mechanism, information interaction between global phenotypic features and local fine-grained features is enhanced, thereby improving the accuracy and robustness of health-status recognition in complex floor-rearing environments.

Specifically, the whole-body region image detected in the first stage is denoted as whole-body, while the comb region image is denoted as comb, and both are used as inputs to the second-stage classification model. For samples containing both whole-body and comb regions, the model adopts a dual-region input mode to jointly model global whole-body features and local fine-grained comb features. For samples containing only a single region due to occlusion, missed detection, or region absence, the model automatically degrades to a single-data-source input mode, utilizing only the available region for health-status recognition. To enable the model to adapt to these different input forms, three types of samples—paired, body-only, and comb-only—are constructed during the training stage, allowing the model to simultaneously handle complete dual-region inputs and single-region inputs. During inference, the input type is automatically determined according to the key-region detection results from the first stage, and the corresponding recognition pathway is selected adaptively.

During the feature extraction stage, the whole-body and comb images are fed into separate feature extraction branches to obtain global whole-body phenotypic features and local key comb features, respectively. Let the whole-body feature be denoted as $f_{b}$ and the comb feature as $f_{c}$. Specifically, $f_{b}$ mainly describes global phenotypic features such as overall appearance, posture, and feather condition, whereas $f_{c}$ mainly represents comb-related characteristics, including color, morphology, and fine-grained abnormal features. Subsequently, the available features are organized into an input sequence X for the Transformer fusion module. For samples containing both whole-body and comb regions, the input sequence can be expressed as:

(1) $$X={[f}_{b},f_{c}]$$

In the feature fusion stage, a Transformer is employed as the core classification network. Different from simple feature concatenation or fixed-weight fusion methods, the Transformer can dynamically model the correlations among features from different sources through the self-attention mechanism, thereby achieving adaptive fusion between global whole-body features and local comb features. Specifically, the input feature sequence X is first projected through linear mappings into Query (Q), Key (K), and Value (V) representations:

(2) $$Q=XW_{Q},K=XW_{K},V=XW_{V},$$

where Q, K and V denote the query matrix, key matrix, and value matrix, respectively, while $W_{Q}$, $W_{K}$ and $W_{V}$ represent the corresponding learnable weight matrices. The self-attention operation can be formulated as:

(3) $$Attention(Q,K,V)=Softmax(\frac{QK^{T}}{\sqrt{d_{k}}})V,$$

where $K^{T}$ denotes the transpose of the key matrix K, and $QK^{T}$ represents the similarity computation between the query and the transposed key matrices. $d_{k}$ indicates the dimensionality of the key vectors, and $\sqrt{d_{k}}$ serves as a scaling factor to prevent excessively large dot-product values. The Softmax() is used to convert the similarity scores into attention weights. Through this process, the model can adaptively adjust the contributions of global whole-body features and local comb features in health-status recognition based on their correlations within the current sample. After Transformer-based fusion, a joint whole-body–comb representation $f_{\mathrm{fusion}}$ is obtained and fed into a fully connected classification layer to output the predicted health status of the chicken:

(4) $$\hat{y}=Classifier(f_{fusion}),$$

where $\hat{y}$ denotes the predicted health-status category, which is divided into two classes: healthy and unhealthy. Through the above design, the proposed model can adaptively perform health-status recognition under both complete dual-region inputs and single-region inputs, thereby improving the stability and robustness of yellow-feathered broiler health-status recognition in complex floor-rearing environments.

### 2.3. Experimental Setup

The experiments in this study were conducted on Ubuntu 20.04. The hardware platform consisted of an Intel Core i5-12400F CPU with a maximum clock frequency of 4.8 GHz, 16 GB of RAM, and an NVIDIA RTX 4090 GPU with 24 GB of memory. The proposed models were implemented using the PyTorch 2.4.1+cu124 deep learning framework with Python 3.8. Since the proposed method consists of two stages, namely key-region localization and health-status recognition, different training parameters were configured for each stage. For the first-stage key-region localization task, DT-YOLO was trained from scratch without pretrained weights. The model was optimized using stochastic gradient descent with a batch size of 16, a maximum of 300 training epochs, an initial learning rate of 0.01, and a weight decay of 0.0005. The training objective consisted of bounding-box regression loss, binary cross-entropy classification loss, and distribution focal loss, with respective weights of 7.5, 0.5, and 1.5. The checkpoint with the highest validation fitness was selected as the final localization model. For second-stage health-status recognition, a shared ImageNet-pretrained Vision Transformer (ViT-B/16) extracted 768-dimensional features from whole-body and comb images. The features were fused with a learnable fusion token using a two-layer, eight-head Transformer encoder with a 3072-dimensional feed-forward layer and a dropout rate of 0.1. The model was trained using class-weighted cross-entropy loss and the AdamW optimizer. The batch size was set to 8, the number of training epochs was set to 50, the initial learning rate was set to 0.0001, and the weight decay was set to 0.0001. The checkpoint with the highest validation macro-F1 score was retained and evaluated on the independent test set.

Model performance and inference efficiency were evaluated separately for the two stages of the proposed framework. For the first-stage key-region localization task, Precision, Recall, mAP@0.5, mAP@0.5:0.95, model size, and inference speed were used as evaluation metrics. Precision and Recall were calculated for the chicken and comb categories to assess the correctness and completeness of region localization. The mAP@0.5 value denotes the mean average precision at an intersection-over-union (IoU) threshold of 0.50, whereas mAP@0.5:0.95 denotes the mean average precision averaged over IoU thresholds from 0.50 to 0.95 at intervals of 0.05. Model size was reported to quantify parameter-storage overhead. Inference speed was reported as frames per second (FPS), corresponding to the number of original images processed per second. For the second-stage health-status recognition task, unhealthy broilers were defined as the positive class (label 1), whereas healthy broilers were defined as the negative class (label 0). Accuracy was calculated over all test samples. Precision, Recall, and F1-score were first calculated separately for the healthy and unhealthy classes and then averaged to obtain the corresponding macro-averaged metrics. Sensitivity was calculated specifically for the unhealthy class, whereas specificity was calculated for the healthy class. Inference speed was reported as FPS, with each whole-body–comb pair or single-part input configuration counted as one classification sample.

For the second-stage classification task, true positives (TP) denote unhealthy broilers correctly classified as Unhealthy; false positives (FP) denote healthy broilers incorrectly classified as Unhealthy; true negatives (TN) denote healthy broilers correctly classified as Healthy; and false negatives (FN) denote unhealthy broilers incorrectly classified as Healthy. The evaluation metrics are defined in Equations (5)–(11).

(5) $$Accuracy=\frac{TP+TN}{TP+TN+FP+FN}$$

(6) $$Precision=\frac{TP}{TP+FP}$$

(7) $$Recall=\frac{TP}{TP+FN}$$

(8) $$F1-score=2\times \frac{Precision\times Recall}{Precision+Recall}$$

(9) $$Sensitivity=\frac{TP}{TP+FN}$$

(10) $$Specificity=\frac{TN}{TN+FP}$$

(11) $$Accuracy=\frac{TP+TN}{TP+TN+FP+FN}$$

For the positive class, Recall and Sensitivity are mathematically equivalent because both quantify the proportion of actual unhealthy broilers that are correctly identified. Therefore, Sensitivity was reported specifically for the Unhealthy class.

## 3. Results

In this section, we will focus on discussing the performance results of the proposed framework.

### 3.1. Results of Key-Region Localization

#### 3.1.1. Performance Comparison with Mainstream Object Detection Models

To validate the effectiveness of the proposed DT-YOLO model for simultaneous key-region localization of chickens in complex floor-rearing environments, comparative experiments were conducted against several mainstream object detection models, including YOLO-v3tiny [32], YOLO-v5 [33], YOLO-v8, YOLO-v12 [34], and RT-DETR(Real-Time DEtection TRansformer) [35]. All models were trained and tested under the same dataset splits, input resolutions, and training configurations to ensure fair comparison. Table 1 presents the performance comparison of the different object detection models on the test set.

Compared with several mainstream object detection models, the proposed DT-YOLO achieved the best overall performance across all evaluation metrics. Specifically, DT-YOLO obtained a Precision of 96.52%, Recall of 97.15%, mAP@0.5 of 97.56%, and mAP@0.5:0.95 of 79.15%, representing improvements of 12.90%, 17.29%, 12.78%, and 20.96%, respectively, over the baseline YOLO-v8 model. In addition, DT-YOLO outperformed recent advanced detectors, including YOLO-v12 and RT-DETR, demonstrating its superior capability for chicken and comb localization in complex floor-rearing environments. These results indicate that the proposed method effectively enhances both detection accuracy and localization performance while maintaining a moderate model size of 22.67 M parameters and a competitive inference speed of 43.58 FPS.

Among the compared models, RT-DETR achieved relatively strong performance, with an mAP@0.5 of 92.23%; however, its parameter count reached 42.02M, which was nearly twice that of DT-YOLO. In contrast, DT-YOLO achieved higher detection accuracy with fewer parameters, indicating a better balance between performance and model complexity. The performance gains can be attributed to the improved feature extraction and representation capabilities introduced by the proposed framework. Specifically, the detection branch enhances the localization of small-scale comb regions under low-light and occlusion conditions, while the adaptive fusion strategy further exploits complementary information from global chicken appearance and local comb features. Consequently, DT-YOLO exhibits stronger robustness and discrimination ability in real-world poultry farming scenarios.

#### 3.1.2. Ablation Study of the Proposed DT-YOLO Model

To assess the contribution of each component in DT-YOLO, ablation experiments were performed based on YOLO-v8. Data Augmentation (DA), RevCol, and InvSE were sequentially incorporated into the baseline model. As shown in Table 2, Groups A–D represent the baseline model, DA-enhanced model, DA+RevCol model, and the final DT-YOLO model with InvSE, respectively.

As shown in Table 2, the baseline model (Group A) achieved mAP@0.5 values of 89.14% and 80.42% for the chicken and comb categories, respectively. These results indicate that YOLO-v8 exhibits satisfactory detection capability for the overall chicken region. However, its performance remains limited when detecting small-scale local targets such as combs. This limitation is mainly attributed to the small size of comb regions in practical floor-rearing environments, where low illumination, occlusion, and background interference frequently occur, making it difficult for the network to effectively capture fine-grained features related to comb color, contours, and morphological characteristics. After introducing the data augmentation strategy, Group B achieved improvements of 2.61%, 9.99%, and 9.48% in Precision, Recall, and overall mAP@0.5, respectively. These results suggest that the expanded sample distribution enhances the model’s adaptability to complex floor-rearing environments and improves its robustness against variations in illumination, posture, and occlusion. With the further incorporation of RevCol, Group C exhibited additional increases of 3.60% in Precision and 1.57% in overall mAP@0.5. This finding indicates that RevCol strengthens cross-level feature interaction and information propagation, thereby improving regional feature representation and enhancing the model’s perception capability under occluded conditions. Finally, after integrating the InvSE module, the Precision, Recall, and overall mAP@0.5 of Group D increased to 96.52%, 97.15%, and 97.56%, respectively. These results demonstrate that the InvSE module effectively enhances discriminative feature representations associated with comb color, morphology, and edge textures. By encouraging the network to focus on informative local regions while suppressing irrelevant background responses, the proposed module further improves the detection performance of comb regions under low-light conditions.

From the category-level detection results, the mAP@0.5 values of the chicken and comb categories improved by 9.11% and 16.45%, respectively, with the progressive integration of the proposed modules. The larger performance gain observed for the comb category suggests that the proposed strategy is particularly effective for small-scale targets and can alleviate the localization challenges caused by low-light conditions. Compared with the overall chicken region, comb regions contain fewer visual cues and finer structural details, making them more dependent on effective feature extraction. By enhancing cross-level feature interaction and emphasizing discriminative local information, the combination of RevCol and InvSE appears to improve comb detection performance under the evaluated conditions. As a result, DT-YOLO is able to provide more reliable regional representations for the adaptive fusion framework in the subsequent stage.

#### 3.1.3. Visual Comparison of Detection Results in Typical Complex Scenes

**Figure 6:**
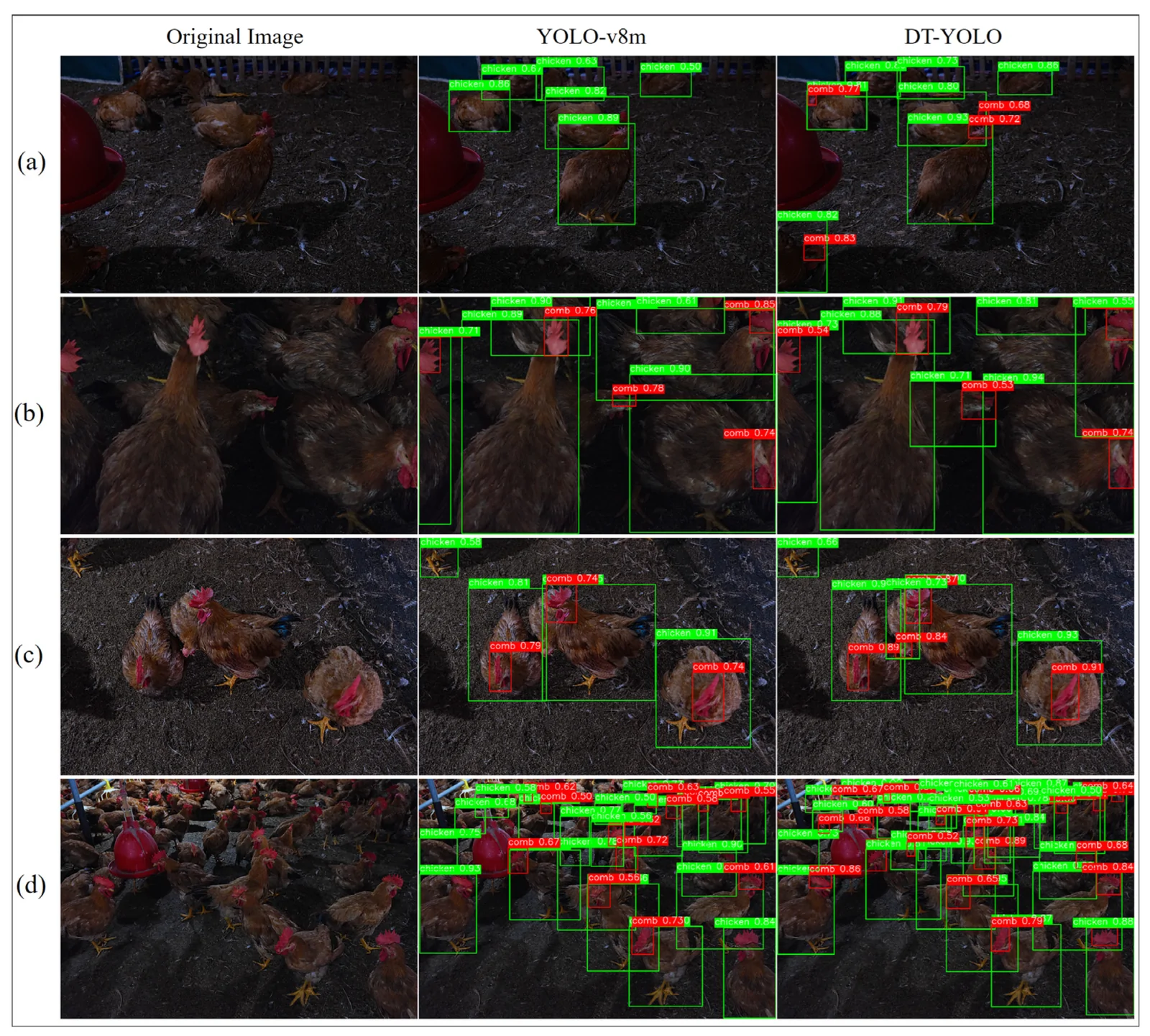
Visual comparison of detection results between YOLO-v8m and DT-YOLO in typical complex floor-rearing scenes: (**a**) low illumination; (**b**) partial occlusion; (**c**) severe occlusion; (**d**) dense multi-target scene.

To further compare DT-YOLO with the baseline YOLO-v8m, qualitative analysis was conducted under representative challenging scenarios, including low illumination, mild occlusion, severe occlusion, and dense target distributions. Figure 6 presents the detailed detection results. By visually comparing the localization results of chicken and comb regions, the effectiveness of the proposed improvements in improving feature representation and key-region perception under complex conditions can be intuitively demonstrated.

Figure 6 provides a qualitative comparison of YOLO-v8m and DT-YOLO under several challenging conditions. In the low-illumination example (Figure 6a), YOLO-v8m missed several chickens in shadowed areas, whereas DT-YOLO localized more of the visible chicken and comb regions. Under mild occlusion (Figure 6b), YOLO-v8m detected some salient comb regions but failed to localize parts of the corresponding chicken bodies, while DT-YOLO produced more complete localization results. In the severe-occlusion example (Figure 6c), DT-YOLO identified more visible targets than YOLO-v8m despite the limited image information. Similarly, in the dense multi-target scene (Figure 6d), DT-YOLO showed fewer apparent missed detections. These examples qualitatively illustrate the potential advantages of DT-YOLO in challenging floor-rearing scenes; however, they do not constitute a separate quantitative analysis of low illumination or occlusion robustness.

### 3.2. Results of Health Status Recognition

#### 3.2.1. Effectiveness of Whole-Body and Comb Fusion

To evaluate the effectiveness of whole-body and comb feature fusion for broiler health status recognition, four experimental settings were designed: whole-body only, comb only, whole-body + comb with simple concatenation, and whole-body + comb with Transformer fusion. The first two settings assess the recognition capability of individual regions, while the latter two compare different fusion strategies. Transformer fusion corresponds to the proposed global–local feature fusion framework. For a fair comparison, paired whole-body and comb samples from the same individual were used in all experiments. The results are presented in Table 3.

To assess the contributions of global and local visual cues, ablation experiments were performed using whole-body images, comb images, and their combination. As shown in Table 3, the whole-body-only model achieved an accuracy of 94.80%, with Macro-Precision, Macro-Recall, and Macro-F1 scores of 94.65%, 94.41%, and 94.53%, respectively. Its sensitivity for unhealthy broilers was 95.19%, whereas its specificity for healthy broilers was 92.65%. In comparison, the comb-only model yielded lower values for accuracy (89.01%), Macro-Precision (88.27%), Macro-Recall (89.40%), and Macro-F1 (88.68%). Its sensitivity decreased to 87.62%, although its specificity was 91.18%. These results suggest that whole-body images provide more comprehensive health-related information, including posture, body contour, plumage condition, and other observable characteristics, while comb features provide useful but relatively limited information when used alone.

To further investigate whether integrating whole-body and comb information can improve recognition performance, two fusion strategies were evaluated. When both regions were available, simple concatenation achieved an accuracy of 93.60% and a Macro-F1 score of 93.59%, which were 1.20 and 0.94 percentage points lower, respectively, than those of the whole-body-only model. Its sensitivity was 93.33%, while its specificity reached 97.06%. In contrast, Transformer-based fusion achieved the highest performance, with an accuracy of 97.11%, Macro-Precision of 97.38%, Macro-Recall of 96.58%, and Macro-F1 of 96.95%. Compared with the whole-body-only model, the Transformer-based approach improved accuracy, Macro-Precision, Macro-Recall, and Macro-F1 by 2.31, 2.73, 2.17, and 2.42 percentage points, respectively. It also achieved a sensitivity of 96.84% and a specificity of 97.59%. These findings indicate that modeling interactions between whole-body and comb features may provide more effective use of their complementary information than direct feature concatenation.

The confusion matrix of the Transformer-fusion model is presented in Table 4. Among the 326 paired samples, 162 healthy broilers and 153 unhealthy broilers were correctly classified. Four healthy broilers were incorrectly classified as unhealthy, and five unhealthy broilers were incorrectly classified as healthy. These nine misclassifications may be associated with low-light imaging conditions or relatively weak visual manifestations in the whole-body and comb regions. Overall, the results indicate that the joint use of whole-body and comb information may support the visual screening of unhealthy broilers, although a small number of ambiguous cases remain.

#### 3.2.2. Comparison with Representative Dual-Input Classification Models

To further validate the effectiveness of the proposed dual-input fusion classification model, four representative classification networks, namely VGG-16 [36], ResNet-18 [37], EfficientNet-V2 [38], and MobileNet-V3 [39], were selected as comparison models. Experiments were conducted using paired whole-body and comb samples as inputs. For each comparison model, the whole-body and comb images were fed into two separate feature extraction branches. The extracted global whole-body features and local comb features were then fused and passed to a classification layer to predict the health status of the chickens. The experimental results are presented in Table 5.

As shown in Table 5, EfficientNet-V2 achieved the highest accuracy among the comparison models (95.95%), followed by ResNet-18 (95.69%), MobileNet-V3 (93.06%), and VGG-16 (91.91%), respectively. The relatively strong performance of ResNet-18 and EfficientNet-V2 may be related to the use of residual connections and efficient scaling strategies, which can enhance feature extraction capability. However, these architectural properties were not independently analyzed in this study. EfficientNet-V2 also obtained the highest Macro-F1 score among the baseline models (95.81%), whereas VGG-16 showed the lowest overall performance. Regarding inference speed, VGG-16 and ResNet-18 achieved higher throughput than EfficientNet-V2, while MobileNet-V3 provided a relatively lightweight alternative with a speed of 62.76 pairs/s.

The proposed model achieved the highest accuracy (97.11%), Macro-Precision (97.38%), and Macro-F1 score (96.95%) among the evaluated models. Compared with EfficientNet-V2, which provided the strongest baseline performance, the proposed model improved Accuracy, Macro-Precision, and Macro-F1 by 1.16, 1.99, and 1.14 percentage points, respectively. It also yielded a 1.47-percentage-point higher sensitivity for unhealthy broilers and a 3.30-percentage-point higher specificity for healthy broilers. Although its inference speed (75.82 pairs/s) was lower than those of VGG-16 and ResNet-18, it was higher than that of EfficientNet-V2 and MobileNet-V3. These results suggest that the proposed Transformer-based fusion model can provide a favorable balance between classification performance and inference efficiency for paired whole-body–comb inputs.

To examine the robustness of the proposed model under incomplete visual information, its performance was evaluated under four input-availability conditions: head-excluded body-only inputs, comb-only inputs, paired whole-body–comb inputs, and mixed input types.

As shown in Table 6, the model achieved an Accuracy of 85.23% and a Macro-F1 score of 81.19% with head-excluded body-only inputs, while comb-only inputs yielded slightly higher values of 86.56% and 83.68%, respectively. When both whole-body and comb inputs were available, performance improved to 97.11% Accuracy and 95.95% Macro-F1. Under the mixed-input condition, the model maintained comparable performance, with an Accuracy of 96.06% and a Macro-F1 score of 96.02%. These results suggest that the proposed model can make use of available visual cues under incomplete-input conditions, with the observed performance variation remaining within an acceptable range for preliminary robustness assessment.

#### 3.2.3. Model Interpretability via Attention Map Visualization

**Figure 7:**
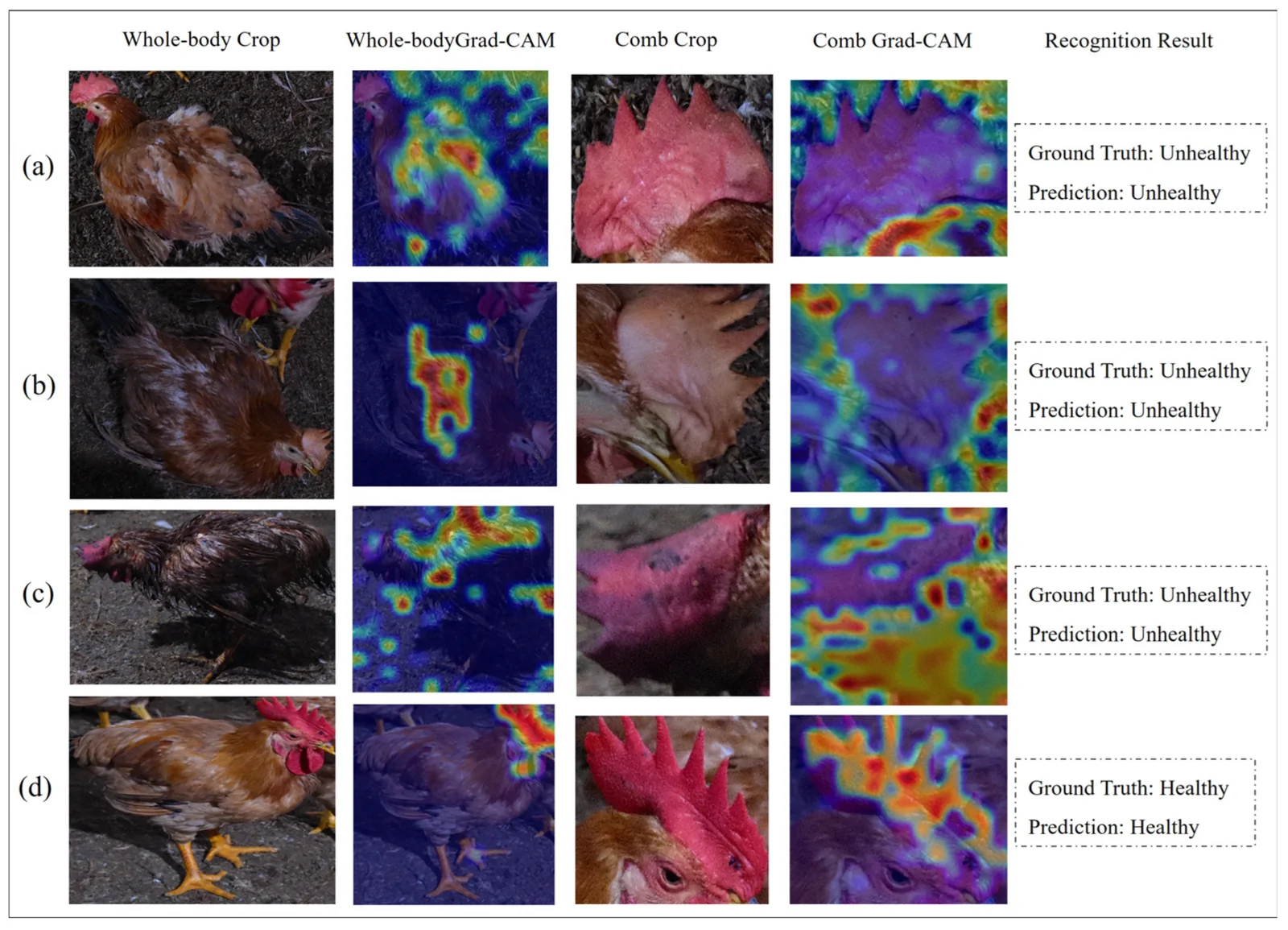
Visualization of health status recognition results using whole-body and comb attention maps: (**a**) body-supported unhealthy; (**b**) comb-responsive unhealthy; (**c**) dual-cue unhealthy; (**d**) healthy.

To further explore the interpretability of the proposed dual-input fusion model, class activation map (CAM) visualization was performed on representative samples from different health-status categories [40]. As shown in Figure 7, four typical recognition scenarios were selected, including body-supported unhealthy, comb-responsive unhealthy, dual-cue unhealthy, and healthy. For each sample, the whole-body image, whole-body CAM, comb image, comb CAM, and final prediction result are presented. By visualizing the spatial distribution of model attention, the contribution of global whole-body characteristics and local comb features to the final classification decision can be intuitively analyzed, thereby providing insight into the model’s decision-making process.

Figure 7a shows an unhealthy broiler whose comb appears relatively normal, whereas the plumage is disordered and the overall body appearance is atypical. The whole-body CAM displays higher activation in parts of the body region corresponding to these visible characteristics, suggesting that the model used global phenotypic information in this example. Figure 7b presents another unhealthy broiler with relatively unremarkable body condition but apparent comb drooping and color variation. In this case, the comb CAM shows higher activation in the comb region, indicating that local comb information contributed to the model output. These examples illustrate that the relative contribution of the whole-body and comb inputs may vary according to the visible characteristics of individual samples.

Figure 7c shows an unhealthy broiler with apparent changes in both regions, including comb drooping and color variation together with disordered or wet plumage. Both CAMs show activation in parts of the corresponding whole-body and comb regions, suggesting that the two inputs provided concurrent visual cues for the prediction in this case. Figure 7d presents a healthy broiler with no obvious abnormalities in the plumage or comb. Its CAMs show less concentrated activation than those of the examples in Figure 7a–c, and the prediction agrees with the reference label assigned during expert annotation. Overall, these CAM results provide qualitative evidence that the model attends to different whole-body and comb regions across samples. However, CAM localization alone does not establish the biological significance of these features or their clinical relevance; the visualizations should therefore be interpreted as explanatory observations rather than direct evidence of disease-related mechanisms or diagnostic validity.

## 4. Discussion

The present study evaluated a two-stage framework that combines whole-body and comb information for the visual recognition of unhealthy broilers. Under the paired-input setting, the Transformer-based fusion approach outperformed the single-region, direct-concatenation, and representative backbone-based comparison models evaluated in this study. Existing computer-vision research on poultry health monitoring has addressed several related but distinct tasks. CNN-based classifiers commonly operate on full-frame images or predefined cropped regions to characterize global appearance patterns, whereas YOLO-based methods are primarily designed for individual localization and, in some cases, the recognition of predefined disease-related manifestations. Studies of lameness and gait focus specifically on locomotor impairment and are therefore not directly equivalent to the broader visual identification of unhealthy broilers considered here. In addition, head- and comb-oriented approaches have demonstrated the usefulness of localized color, texture, and morphological cues, but often focus on disease-specific classification or use local regions in isolation. More recent multi-input approaches have highlighted the value of complementary information; however, the input modalities, fusion mechanisms, and target outcomes vary substantially across studies. Against this background, the contribution of the present framework lies in jointly localizing whole-body and comb regions and explicitly modeling their global–local interaction for visual health-status screening. Comparisons with previous studies should nevertheless be interpreted cautiously, as differences in breeds, production systems, health-status definitions, datasets, and evaluation protocols limit direct numerical comparison.

Several limitations related to the dataset and potential commercial use should be acknowledged. The dataset was collected from a single commercial farm and represents one yellow-feathered broiler population, one approximate age range, one floor-rearing system, and a limited set of environmental and management conditions. Consequently, differences in breed, age, sex, plumage characteristics, illumination, stocking density, litter conditions, camera placement, and farm management may alter the visual appearance of both whole-body and comb regions and affect model performance. In addition, health-status labels were assigned through expert assessment of observable phenotypic abnormalities rather than veterinary-confirmed diagnoses. Thus, the framework should be regarded as a tool for screening visually observable abnormalities in broilers, rather than for diagnosing specific diseases or establishing clinical health status. Repeated observations of the same birds may also have occurred because individual broilers could appear in multiple images. Although area-based partitioning was used to reduce the risk of related samples being distributed across subsets, complete independence cannot be ensured without individual identification and tracking. Class imbalance and the absence of an external validation set may further affect the stability and generalizability of the reported results. These factors currently limit conclusions regarding performance in broader commercial applications.

The framework may have potential for adaptation to other commercial poultry settings because it uses externally observable whole-body and comb information that can be acquired with standard imaging equipment. However, its applicability across farms, breeds, lighting conditions, production systems, and management practices remains to be established. Before practical deployment, the model should be evaluated using independent multi-farm datasets that include diverse breeds, age groups, housing systems, seasons, illumination conditions, and management regimes, with farm-, area-, or individual-level separation between training and test data. Future work should also incorporate longitudinal observations and veterinary-confirmed reference labels to determine whether visual changes can be detected consistently over time and to clarify their relationship to specific diseases, stress conditions, or welfare-related states. Extending the current binary visual screening task to multi-class health assessment, uncertainty-aware prediction, and the integration of behavioral, acoustic, environmental, or thermal information may further improve the practical value of the system in commercial farming contexts.

## 5. Conclusions

This study proposed a two-stage framework for the visual recognition of unhealthy yellow-feathered broilers using whole-body and comb information. On the dataset collected from the evaluated commercial floor-rearing farm, the first-stage DT-YOLO model achieved an mAP@0.5 of 97.56% for simultaneous whole-body and comb localization. In the second stage, Transformer-based fusion of paired whole-body and comb inputs achieved an Accuracy of 97.11% and a Macro-F1 score of 96.95%, outperforming the evaluated single-region and direct-concatenation settings. The model also achieved an Accuracy of 96.06% and a Macro-F1 score of 96.02% under the mixed-input condition. These findings suggest that combining global whole-body and local comb information can support the visual screening of unhealthy broilers within the evaluated dataset. However, the framework was developed using data from a single farm and labels based on observable phenotypic abnormalities; its performance across other farms, breeds, management conditions, and veterinary-confirmed health states requires independent external validation.

## Figures and Tables

**Table 1:** Performance comparison of different object detection models.

Model	Precision (%)	Recall (%)	mAP@0.5 (%)	mAP@0.5:0.95 (%)	Parameters (M)	FPS (Images/s)
YOLO-v3tiny	79.20	78.52	79.28	48.99	12.13	60.28
YOLO-v5	80.62	77.50	82.82	53.11	25.05	46.40
YOLO-v8	83.62	79.86	84.78	58.19	25.84	44.40
YOLO-v12	84.24	75.92	84.62	53.29	20.11	48.73
RT-DETR	89.30	90.58	92.23	62.30	42.02	31.82
DT-YOLO	96.52	97.15	97.56	79.15	22.67	43.58

**Table 2:** Ablation study results of the proposed DT-YOLO model.

Group	DA	RevCol	InvSE	Precision (%)	Recall (%)	mAP@0.5 (All)/%	mAP@0.5 (Chicken)/%	mAP@0.5 (Comb)/%
A				83.62	79.86	84.78	89.14	80.42
B	√			86.23	89.85	94.26	95.56	92.96
C	√	√		89.83	86.91	95.83	96.41	95.25
D	√	√	√	96.52	97.15	97.56	98.25	96.87

“√” indicates that the module was added to the method.

**Table 3:** Ablation study on input modalities and fusion strategies on the paired test set.

Input Setting	Fusion	Acc (%)	P_Macro (%)	R_Macro (%)	F1_Macro (%)	Sen_U (%)	Spec_H (%)
whole-body only	—	94.80	94.65	94.41	94.53	95.19	92.65
comb only	—	89.01	88.27	89.40	88.68	87.62	91.18
whole-body+comb	simple concat	93.60	93.66	93.89	93.59	93.33	97.06
whole-body+comb	transformer	97.11	97.38	96.58	96.95	96.84	97.59

Note: Acc, Accuracy; P_Macro, Macro-Precision; R_Macro, Macro-Recall; F1_Macro, Macro-F1 scores; Sen_U, Sensitivity for unhealthy broilers; Spec_H, Specificity for healthy broilers.

**Table 4:** Confusion matrix of the Transformer-based whole-body–comb fusion model.

	Predicted Healthy	Predicted Unhealthy
True Healthy	162	4
True Unhealthy	5	153

**Table 5:** Comparison of representative dual-input classification models on the paired test set.

Model	Acc (%)	P_Macro (%)	R_Macro (%)	F1_Macro (%)	Sen_U (%)	Spec_H (%)	FPS (Pairs/s)
VGG-16	91.91	91.22	92.56	91.67	83.82	98.10	89.48
ResNet-18	95.69	94.37	95.84	95.59	93.44	92.38	88.18
EfficientNet-V2	95.95	95.39	96.41	95.81	95.37	94.29	31.41
MobileNet-V3	93.06	93.21	92.21	92.65	95.59	83.81	62.76
Ours	97.11	97.38	96.58	96.95	96.84	97.59	75.82

Note: Acc, Accuracy; P_Macro, Macro-Precision; R_Macro, Macro-Recall; F1_Macro, Macro-F1 scores; Sen_U, Sensitivity for unhealthy broilers; Spec_H, Specificity for healthy broilers.

**Table 6:** Robustness evaluation on missing-modality test sets.

Input Availability	Samples	Accuracy (%)	Macro F1-Scores (%)
Head-excluded body only	38	85.23	81.19
Comb region only	42	86.56	83.68
Whole-body+comb	326	97.11	95.95
Mixed input types	406	96.06	96.02

## Data Availability

As this is a proprietary dataset belonging to our collaborators, we are unable to disclose it at present.
